# Angina Pectoris Following Latanoprostene Bunod 0.024% Administration: A Case Report

**DOI:** 10.7759/cureus.96522

**Published:** 2025-11-10

**Authors:** Zubair Edakkavil, Misbah Fazlani, Ahmer Longi, Aneez Shaikh, Ravisekhar Sundaram

**Affiliations:** 1 Internal Medicine, Mediclinic Welcare Hospital, Dubai, ARE; 2 Ophthalmology, Mediclinic Welcare Hospital, Dubai, ARE; 3 Cardiology, Mediclinic Welcare Hospital, Dubai, ARE

**Keywords:** adverse side effect, angina pectoris, glaucoma therapy, latanoprostene bunod, ophthalmic drops

## Abstract

Latanoprostene bunod 0.024% is a nitric oxide-donating prostaglandin analog approved for lowering intraocular pressure in patients with open-angle glaucoma and ocular hypertension. Although generally considered safe, systemic adverse effects are rarely reported. We are reporting a case of a 63-year-old healthy Asian male with newly diagnosed normal-tension glaucoma who was prescribed latanoprostene bunod ophthalmic drops, which resulted in him experiencing angina pectoris. On the day of initiation, he experienced throat dryness and heartburn, followed by a sudden episode of intense retrosternal constricting chest pain during the night. A comprehensive cardiological evaluation-including electrocardiography (ECG), cardiac biomarkers, echocardiography, and stress testing-revealed no abnormalities. Notably, the patient’s chest pain resolved completely within 12 hours of discontinuing the eye drops. Although prostaglandin analogs such as latanoprostene bunod are primarily believed to exert localized ocular effects, they may cause systemic reactions through mechanisms including vasoconstriction and nociceptor sensitization-particularly in susceptible individuals. This case underscores the importance of clinical vigilance for atypical systemic symptoms following initiation of topical glaucoma medications and supports the need for further investigation into their cardiovascular safety profile.

## Introduction

Latanoprostene bunod is an ophthalmic solution that contains latanoprostene bunod 0.024%, approved for the reduction of intraocular pressure (IOP) in patients with open-angle glaucoma or ocular hypertension. As a nitric oxide-donating prostaglandin analog, it enhances the outflow of aqueous humor via both the uveoscleral and trabecular meshwork pathways [1]. The physicochemical characteristics of prostaglandin analogs, such as their high lipophilicity and effective permeability across the conjunctival and nasal mucosa, increase the risk of systemic absorption, even with topical application [2]. In previous clinical trials, the overall occurrence of adverse effects over three months was relatively low, with eye irritation and conjunctival hyperemia being the most frequently reported side effects, both of which were generally mild and transient [3]. Prostaglandin F2 alpha (PGF2α) is known for its vasoconstrictive properties. Systemic absorption of latanoprost may induce coronary vasoconstriction, potentially leading to anginal symptoms, particularly in those with unstable coronary artery disease. Additionally, experimental studies in animal models have shown that prostaglandins like PGF2α can trigger cardiac myocyte hypertrophy through the upregulation of genes such as c-fos, atrial natriuretic factor, and α-skeletal actin. This hypertrophic process can elevate myocardial oxygen demand, making patients with compromised coronary circulation more susceptible to ischemia and angina. Although objective electrocardiographic changes, like ST-segment deviations, may not always be evident, clinical manifestations may still align with myocardial ischemia [4]. Because systemic side effects of latanoprostene bunod have rarely been reported, further studies investigating the underlying pathophysiologic mechanism and at-risk populations are necessary.

## Case presentation

**Figure 1:**
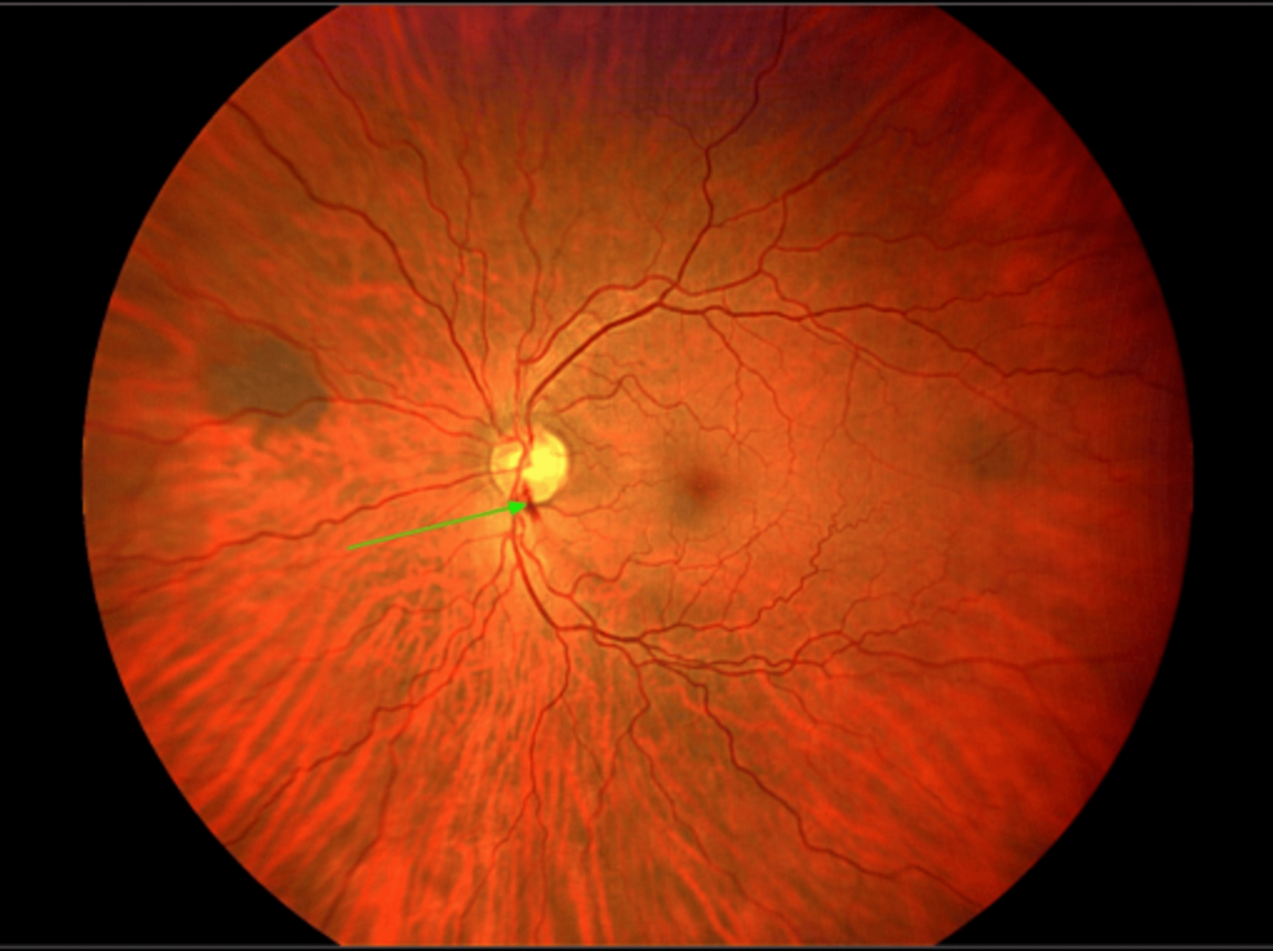
Retinal image demonstrating flame shaped hemorrhage in the retinal nerve fiber layer (green arrow)

A 63-year-old previously healthy Asian man visited the ophthalmology clinic for a routine eye examination and was diagnosed with normal-tension glaucoma. He was referred for a second opinion, where subsequent evaluations corroborated early glaucomatous changes. Ophthalmic evaluation showed bilateral dry eyes, open angles on gonioscopy, normal intraocular pressures, and clear ocular media. Notably, disc hemorrhage with corresponding retinal nerve fiber layer (RNFL) thinning was observed in the left eye (Figure 1). Systemic evaluation was unremarkable. Consequently, he was prescribed latanoprostene bunod ophthalmic solution 0.024%, to be applied as one drop in each eye once daily.

**Figure 2:**
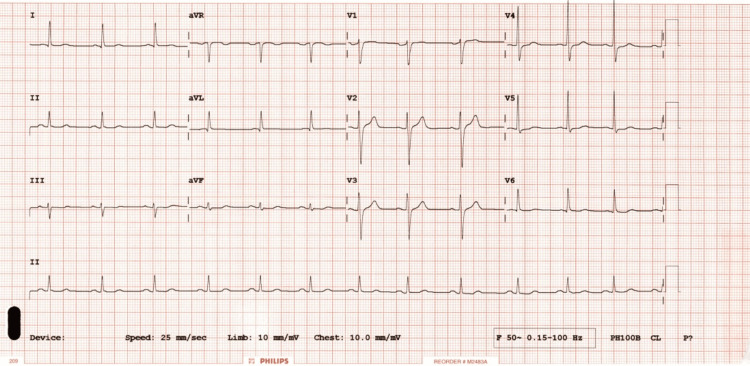
Electrocardiogram on presentation No ST T changes

When the patient started using the eye drops as prescribed, on the first dose itself, he experienced throat dryness and heartburn. He self-treated these symptoms with over-the-counter antacids, providing minimal relief. Later that night, he experienced a sudden and severe retrosternal chest pain that disrupted his sleep. This pain did not respond to additional over-the-counter medications and proton pump inhibitors, prompting him to seek cardiologic evaluation. A thorough cardiac assessment, including electrocardiography (ECG) (Figure 2), serum cardiac enzymes (Table 1), echocardiography, and cardiac stress testing, was performed. All these tests returned normal. Due to ongoing chest discomfort, the patient chose to discontinue the use of the eye drops. Notably, he reported complete resolution of his symptoms in less than 12 hours after stopping the medication.

**Table 1:** Table showing cardiac enzyme value

Laboratory investigation	Result	Reference value
Troponin T	6.69 ng/L	Normal: < 14 ng/L

## Discussion

This case illustrates a rare yet clinically significant adverse event-acute retrosternal chest pain following the use of latanoprostene bunod ophthalmic drops in a patient without a history of cardiovascular disease. While this medication is primarily linked to local ocular side effects like conjunctival hyperemia and eye irritation, systemic effects have been documented in isolated instances, although they remain poorly understood [1, 2].

The temporal correlation between the initiation of the drug and the onset of symptoms, coupled with the complete alleviation of symptoms upon discontinuation, strongly suggests a causal relationship. Although extensive cardiologic assessments-including ECG, cardiac enzymes, echocardiography, and stress testing-yielded normal results, the patient’s clinical presentation was indicative of transient myocardial ischemia.

PGF2α analogues such as latanoprostene bunod possess physicochemical properties (notably high lipophilicity and mucosal permeability) that facilitate systemic absorption following topical administration [3]. Once absorbed systemically, these agents may induce coronary vasoconstriction or affect cardiac myocytes through the upregulation of hypertrophic markers, possibly leading to ischemia in predisposed individuals. Prior literature has also indicated that angina can be exacerbated in patients using latanoprost, suggesting a potential class effect [4]. Although latanoprostene bunod is not contraindicated for patients with stable cardiovascular conditions, this case underscores the importance of exercising caution in individuals with known or suspected coronary vasospasm and encourages clinicians to consider systemic effects when evaluating new-onset chest pain in patients using topical anti-glaucoma medications. The findings here are consistent with previously published reports detailing chest tightness following the use of topical latanoprost, particularly in older patients with possible cardiac vulnerabilities [5]. These findings support the importance of clinical vigilance for possible systemic effects of PGF2α analogues, which include visceral nociceptive and vasoconstrictor influences.

## Conclusions

This case underscores a rare but clinically significant adverse effect of Latanoprostene bunod-induced angina pectoris in a patient without traditional cardiovascular risk factors. The temporal relationship with drug initiation, resolution upon discontinuation, and absence of cardiac pathology suggest a potential class effect among PGF2α analogs. Clinicians should be alert to atypical systemic symptoms, even in low-risk individuals, when initiating topical glaucoma therapy. Further research is warranted to elucidate the underlying mechanisms and better identify vulnerable patient populations.
